# Prior Consumption of a Fat Meal in Healthy Adults Modulates the Brain’s Response to Fat^1^,^2^,^3^

**DOI:** 10.3945/jn.116.234104

**Published:** 2016-09-21

**Authors:** Sally Eldeghaidy, Luca Marciani, Joanne Hort, Tracey Hollowood, Gulzar Singh, Debbie Bush, Tim Foster, Andy J Taylor, Johanneke Busch, Robin C Spiller, Penny A Gowland, Susan T Francis

**Affiliations:** 4 Sir Peter Mansfield Imaging Centre, School of Physics and Astronomy,; 5 Flavour Research Group,; 6 Division of Food Sciences,; 7 School of Biomedical Sciences, and; 8 Nottingham Digestive Diseases Centre and National Institute for Health Research Biomedical Research Unit, Gastrointestinal and Liver Diseases, Nottingham University Hospitals NHS Trust, University of Nottingham, Nottingham, United Kingdom;; 9 Department of Physics, Faculty of Science, Suez Canal University, Ismailia, Egypt;; 10 Division of Surgery, Queen's Medical Centre University Hospital, Nottingham, United Kingdom; and; 11 Unilever, Vlaardingen, Netherlands

**Keywords:** fMRI, BOLD, CBF, CCK, habituation, oral fat, insula, subjective rating satiety

## Abstract

**Background:** The consumption of fat is regulated by reward and homeostatic pathways, but no studies to our knowledge have examined the role of high-fat meal (HFM) intake on subsequent brain activation to oral stimuli.

**Objective:** We evaluated how prior consumption of an HFM or water load (WL) modulates reward, homeostatic, and taste brain responses to the subsequent delivery of oral fat.

**Methods:** A randomized 2-way crossover design spaced 1 wk apart was used to compare the prior consumption of a 250-mL HFM (520 kcal) [rapeseed oil (440 kcal), emulsifier, sucrose, flavor cocktail] or noncaloric WL on brain activation to the delivery of repeated trials of a flavored no-fat control stimulus (CS) or flavored fat stimulus (FS) in 17 healthy adults (11 men) aged 25 ± 2 y and with a body mass index (in kg/m^2^) of 22.4 ± 0.8. We tested differences in brain activation to the CS and FS and baseline cerebral blood flow (CBF) after the HFM and WL. We also tested correlations between an individual’s plasma cholecystokinin (CCK) concentration after the HFM and blood oxygenation level–dependent (BOLD) activation of brain regions.

**Results:** Compared to the WL, consuming the HFM led to decreased anterior insula taste activation in response to both the CS (36.3%; *P <* 0.05) and FS (26.5%; *P <* 0.05). The HFM caused reduced amygdala activation (25.1%; *P <* 0.01) in response to the FS compared to the CS (fat-related satiety). Baseline CBF significantly reduced in taste (insula: 5.7%; *P <* 0.01), homeostatic (hypothalamus: 9.2%, *P <* 0.01; thalamus: 5.1%, *P <* 0.05), and reward areas (striatum: 9.2%; *P <* 0.01) after the HFM. An individual’s plasma CCK concentration correlated negatively with brain activation in taste and oral somatosensory (ρ = −0.39; *P* < 0.05) and reward areas (ρ = −0.36; *P* < 0.05).

**Conclusions:** Our results in healthy adults show that an HFM suppresses BOLD activation in taste and reward areas compared to a WL. This understanding will help inform the reformulation of reduced-fat foods that mimic the brain’s response to high-fat counterparts and guide future interventions to reduce obesity.

## Introduction

Food consumption is regulated by both hedonic and homeostatic pathways. The hedonic decrease in the pleasantness of a given eaten food, termed “sensory-specific satiety,” has been found to play an important role in food choice and meal termination (1). Homeostatic regulation via circulating gut peptide hormones, including cholecystokinin (CCK)^13^^13^ (2), YY_3–36_ (3), and glucagon-like peptide 1 (4), also plays a key role in food intake by communicating with the central nervous system to provide feedback to control energy demand and the cessation of feeding. CCK is stimulated by the presence of fat in the small intestine; this stimulation acts on receptors in the vagus nerve (2) and activates the brainstem nucleus tractus solitarius, which projects to brain regions that control food intake (e.g., hypothalamus, thalamus, and striatum) (5). A small number of fMRI studies have assessed brain activation to oral fat samples in humans and have shown fat to activate taste, texture, and reward areas (6–9). fMRI studies in healthy human subjects have also assessed the brain’s response to an intravenous (10) or intragastric (11, 12) infusion of gut hormones, thus eliminating the sensation of taste associated with the oral consumption of food. These studies have shown a YY_3–36_ (10) and CCK (11) concentration–dependent increase in blood oxygenation level–dependent (BOLD) signal in homeostatic regions of the brainstem and hypothalamus and hedonic reward areas of the orbitofrontal cortex (OFC) and anterior cingulate cortex (ACC). A few fMRI studies have shown that the oral consumption of food to satiety decreases the BOLD signal in reward areas, including the amygdala and OFC (13–15).

Our objective was to use fMRI to study the modulation of the reward, homeostatic, and taste brain responses to an oral fat stimulus (FS) and control stimulus (CS) after the prior oral consumption of a high-fat meal (HFM) compared to a water load (WL). We hypothesized that *1*) the prior consumption of an HFM would modulate the subsequent BOLD response to oral stimulation provided by the same FS (fat-related satiety) in reward, taste, and homeostatic areas (16); *2*) baseline cerebral blood flow (CBF) would be reduced in areas involved in reward, appetite regulation, and taste after an HFM compared to a WL; and *3*) individuals’ CCK concentrations would correlate with the amplitudes of their BOLD responses to the CS and FS in areas involved in reward, appetite, and taste.

## Methods

### Participants

Participants for this study included 17 healthy right-handed individuals (11 men) aged 25 ± 2 y with a BMI (in kg/m^2^) of 22.4 ± 0.8. Volunteers were screened for taster status by rating the intensity of 6-*n-*propylthiouracil (PROP) on the general labeled magnitude scale (17). To ensure the statistical power in the fMRI response, subjects took part in the study if they were classified as a PROP taster (10 subjects were super tasters and 7 medium tasters) (7). Classifying subjects as tasters and nontasters according to their fat sensitivity was not performed because rapeseed oil, a TG with no free FAs and more textual perception than taste, was used in the samples in this study.

Subjects were asked to consume a nonfatty dinner the evening before the study and a light breakfast on the day of the study and then to fast for ≥2 h (no food and no caloric or caffeine-containing beverages) before each scan. On each scan day, the subject’s forearm vein was cannulated for serial venous blood sampling from which to measure the plasma CCK concentration. The CCK concentration at baseline also verified subject compliance to eating restrictions.

### Stimuli and delivery system

**FIGURE 1:**
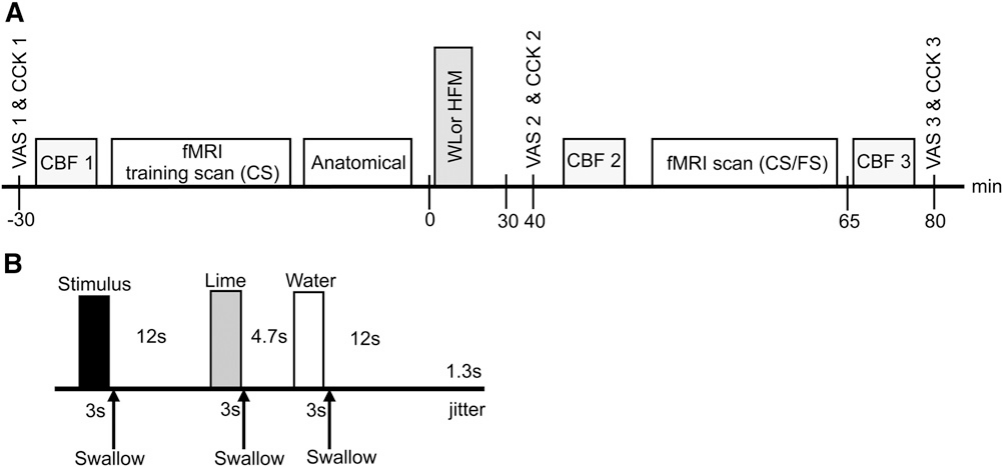
Overall design of study day (A) and a single fMRI trial (B). The WL and HFM conditions had the same design. During the fMRI scan, 18 trials of both the CS and FS were delivered in a pseudorandomized order. CBF, cerebral blood flow; CCK, cholecystokinin; CS, control stimulus; FS, fat stimulus; HFM, high-fat meal; VAS, visual analog scale; WL, water load.

Two emulsion stimuli were delivered during a block-design fMRI paradigm (**Figure 1**), a flavored no-fat CS and a flavored FS. The composition and caloric values of the CS and FS samples are provided in **Supplemental** **Tables** **1** and **2**. The FS matched the HFM and was designed to have the same physiochemical effects [perceived fruitiness, sweetness, and viscosity (isoperceived)] as the CS. To match the perceived fruitiness intensity between the 2 stimuli, the composition of the flavor cocktail was increased 3-fold more for the FS compared to the CS based on a full sensory design space (18).

Stimuli were delivered with the use of a reproducible automated spray system via nozzles positioned in the middle of the subjects’ mouths to ensure the dispersion of stimuli across the tongue and mouth surfaces (19). A visual cue instructed the subjects to swallow 3 s after the onset of stimulus delivery. Surface electromyography was acquired concurrently with the fMRI data and processed with the use of a BrainVision Analyzer (Brain Products) (7) to estimate the exact time subjects swallowed, thus allowing the duration of each stimulus within the mouth to be modeled.

### Study protocol

A randomized 2-way crossover design was used to assess the effect of the prior consumption of either a high-caloric HFM or noncaloric WL on the BOLD response to the oral CS and FS. Subjects took part in 2 scan sessions ≥1 wk apart and consumed 250 mL of either WL or HFM (Figure 1). The WL was chosen as a neutral meal of equal volume to the HFM, as used in Spetter et al. (12), although the viscosity and energy content did not match the HFM (12).

All subjects first underwent an fMRI training scan during which only the CS was delivered to subjects. This familiarized subjects to the scan environment before the consumption of either the HFM or WL. Subjects then came out of the scanner and sat in a quiet room and consumed 250 mL of either the HFM or WL within a 15-min period from a cup (without a straw). After 45 min, when the CCK concentration was predicted to be raised, subjects took part in an fMRI scan to assess the BOLD activation to the CS and FS.

The fMRI training scan consisted of 3 mL CS delivered over a 3-s period with a 1-mL/s flow rate. Twelve seconds after cessation of the CS delivery, 2 mouth rinses were given, the first of which comprised 3 mL 3.5% wt:wt lime juice in still mineral water (lime) and the second of which, delivered 4.7 s later, comprised 3 mL still mineral water (water); each mouth rinse was delivered over a 3-s period. Subjects were visually cued to swallow immediately at the end of each stimulus delivery to give a clearly defined stimulus bolus period. This reduced variations in stimulus properties caused by different interactions with the saliva and ensured natural physiologic retronasal aroma stimulation (19, 20). A delay of 12 s followed the second mouth rinse; this entire trial was then repeated. In total, 18 fMRI trials were collected, during which 54 mL CS was delivered in ∼10 min. All stimuli and rinse solutions were delivered at room temperature.

The fMRI scan protocol was similar to the fMRI training scan, but both CS and FS were delivered in a random order. This procedure was repeated for 36 trials (18 trials of each stimulus) and lasted ∼20 min.

Subjective satiety ratings (fullness, hunger, and appetite) and blood samples from which to estimate plasma CCK concentration were collected at 3 time points: in a fasted state before the fMRI training scan (baseline), before the fMRI scan (30 min postmeal), and after the fMRI scan (80 min postmeal) (Figure 1). In addition, arterial spin labeling (ASL) measures of CBF were collected at baseline and at 40 and 65 min postmeal (Figure 1), and an anatomic image was acquired after the fMRI training scan.

### Subjective ratings

Subjective ratings of fullness, hunger, and appetite (desire to eat) were assessed on a self-assessment visual analog scale. Subjects were instructed to mark on a 100-mm continuous line *1*) how full they felt, *2*) how hungry they felt, and *3*) how much food they could eat.

### CCK measurement

Blood samples were transferred into collection tubes containing 0.3 mL EDTA and 5000 KIU aprotinin. The plasma was then separated by centrifugation at 4°C and stored at −80°C until assessed. Plasma samples were extracted before being assayed to eliminate nonspecific interference from plasma proteins. CCK concentration was measured by RIA (EURIA CCK; Euro-Diagnostica). The minimum detectable CCK concentration was 0.3 pmol/L.

### MRI scanning

Scanning was performed on a 3.0 Tesla Philips Achieva scanner with an 8-element SENSE head coil. fMRI data were collected with the use of a double-echo gradient echo-echo-planar imaging (GE-EPI) acquisition: echo time (TE) = 30/49 ms; repetition time (TR) = 2600 ms (jittered); and 36 continuous transverse slices with a 4-mm isotropic spatial resolution (64 × 64 matrix). The double-echo acquisition provided increased BOLD sensitivity in areas with a short T_2_* (7, 19) such as the amygdala and hypothalamus, which are particularly important in reward and feeding. After fMRI acquisition, a multiecho EPI data set (TE: 11, 30, 49, 68, and 87 ms; TR: 10 s) was acquired with matched geometry to the fMRI data.

CBF data were collected with the use of a QUIPSS II FAIR ASL scheme (21). The labeling scheme comprised a 54-mm selective inversion slab and 250-mm spatially limited nonselective inversion slab, with in-plane saturation with the use of water suppression-enhanced through T_1_ effects presaturation and sinc postsaturation. ASL data were acquired with a GE-EPI readout of identical spatial resolution and bandwidth to the fMRI acquisition; however, a subset of 11 slices of the fMRI data set (centered on the anterior insula) was acquired with minimal temporal spacing and a reduced TE of 14 ms. All ASL data were collected at a postlabel delay of 1550 ms with an ASL pair collected in a TR_ASL_ of 6 s. In addition, an equilibrium magnetization image was acquired with a long TR for CBF quantification. An MPRAGE anatomic image (1-mm isotropic resolution; linear phase-encoding order; TE/TR = 3.7/8.1 ms; flip angle = 8° inversion time = 960 ms; 256 × 256 matrix) was also acquired. Each study session lasted ∼2 h and included MRI data acquisition, blood sampling, collection of subjective visual analog scale ratings, and the time to consume the WL/HFM.

### Data and statistical analyses

Tests of normality and statistical differences were performed with the use of SPSS software version 14 (IBM). Values are presented as means ± SEMs or medians (IQRs). *P* ≤ 0.05 was considered significant.

### Subjective ratings analysis

Subjective ratings of hunger, fullness, and appetite were tested for normality with the use of the Shapiro-Wilk normality test. All subjective ratings were normally distributed, and the mean of each rating was calculated at baseline (30 min premeal) and at 30 and 80 min postmeal (Figure 1). A paired *t* test was used to assess any significant differences in subjective ratings between the WL and HFM at each of the 3 time points. In addition, a Kruskal-Wallis test was performed to assess whether subjective ratings changed significantly over the 3 time points for the WL and HFM.

### CCK analysis

Plasma CCK concentrations were tested for normality with the use of the Shapiro-Wilk normality test and were found to be nonnormal; thus, the median value was estimated at each time point. A nonparametric 2-tailed Wilcoxon test was used to assess any significant difference in CCK plasma concentration between the WL and HFM at each time point. A Kruskal-Wallis test was performed to assess the significance of change between the 3 time points for a given meal. In addition, a Spearman correlation (ρ) test was performed for each subject to test the strength of the relation between each subjective rating and CCK concentration across the 3 time points (baseline and at 30 and 80 min postmeal).

### fMRI data analysis

fMRI data sets were processed with the use of statistical parametric mapping software (SPM5; Welcome Department of Cognitive Neurology). Slice-timing correction and realignment were applied to the double-echo GE-EPI images. Individual realignment parameters were inspected to ensure no subject moved by >1 voxel during the fMRI scan. T_2_* maps were formed from the multiecho data set with the use of a voxel-by-voxel, linear, weighted least-squares fit and used in the weighted summation of the double-echo fMRI data. The weighted fMRI data were spatially normalized to the Montreal Neurological Institute (MNI) template and spatially smoothed with an 8-mm full-width-at-half-maximum isotropic Gaussian kernel.

A first-level general linear model analysis was performed with the use of the time each CS or FS sample remained in the mouth, as calculated from the electromyography trace, convolved with a canonical hemodynamic response function. Data were globally scaled and temporally filtered with an 80-s high-pass filter cutoff. The 2 mouth-rinse events and motion parameters were included as conditions and covariates, respectively, of no interest.

To identify the brain activation to each oral stimulus after the consumption of the HFM/WL, 4 contrast vectors were formed: CS (compared to rest) after the HFM, FS (compared to rest) after the HFM, CS (compared to rest) after the WL, and FS (compared with rest) after the WL. Each of these first-level CS and FS brain activation maps was pooled in a second-level random-effects (RFX) group analysis and brain activation displayed at a false-discovery rate–corrected probability of *P* > 0.05, with a cluster of >10 voxels considered significant. This brain activation reflects the response to all sensory attributes of the stimulus: viscosity, taste, fat content, flavor, and spray delivery and swallow. We then performed statistical analyses to assess *1*) the effect of prior consumption of an HFM and WL on BOLD activation; *2*) the effect of prior consumption of an HFM and WL on the trial-by-trial BOLD response; *3*) the alteration in CBF in response to the HFM and WL; and *4*) the effect of increasing subjective plasma CCK concentration after the HFM on the BOLD response. These respective analyses are discussed in the 4 subsections that follow.

#### Effect of prior consumption of an HFM and WL on BOLD activation.

We used a paired *t* test to compare brain activation to the CS and FS after the HFM with the WL (contrasts of HFM > WL and WL > HFM). For these differential contrasts, a binary mask of the BOLD activation to both the CS and FS threshold of *P* ≤ 0.05 (uncorrected) was used to define a priori areas of interest. Differential contrast maps were corrected with the use of small-volume correction in an 8-mm radius sphere around a peak voxel defined a priori from relevant areas reported in fMRI studies of fat emulsions (7, 18); these included taste and aroma (anterior insula), somatosensory [primary and secondary somatosensory cortexes (SI and SII), middle and posterior insula], reward (amygdala, ACC, OFC), and homeostatic areas (thalamus, hypothalamus, striatum).

A region-of-interest (ROI) analysis based on a priori areas was also performed on each individual subject’s data. Three 8-mm spherical ROIs centered at MNI coordinates (40, 10, −2), (40, 0, 0), and (44, −32, 12) were used to assess the anterior, middle, and posterior insula, respectively (7). The hypothalamus was defined as an 8-mm sphere radius centered at (0, −4, −8), the peak voxel reported by Smeets et al. (15). The mid-OFC (−6, 44, −2) and lateral OFC (26, 32, −10) coordinates were defined as an 8-mm sphere radius centered at the peak voxel reported by de Araujo and Rolls (6). The thalamus and amygdala were anatomically defined by the PickAtlas. The ROIs contained a large number of voxels (>250), encompassing all active voxels in each area of interest and allowing for variability in the location of the activation peak within cortical regions across subjects. For each ROI, the mean of the top 5% of BOLD β-value parameter estimates was assessed (18). Because all ROIs had a large number of voxels, this analysis approach ensured that the activity in each functional area could be assessed with a high signal-to-noise ratio while still accounting for any between-subject functional variability (e.g., because of differences in cortical folding patterns). Because these ROIs were defined based on a priori hypotheses, *t* tests were not corrected for multiple comparisons. To assess fat-related satiety, a paired *t* test was performed to compare brain activation to the FS after the HFM to the WL.

#### Effect of prior consumption of an HFM and WL on the trial-by-trial BOLD response.

To assess the habituation and enhancement of BOLD activation to the CS and FS across repeated fMRI trials, a linear parametric modulation in time was included in the general linear model. Brain areas that showed a significant trial-by-trial decrease or increase in the BOLD signal were identified. For this analysis, a binary mask of CS and FS was used. *P* ≤ 0.001 (uncorrected and corrected with the use of small-volume correction) was considered the threshold of significance. To further illustrate the trial-by-trial modulation, the BOLD percentage signal change was plotted against fMRI trial number (mean across subjects) in selected a priori ROIs, and a linear regression analysis and Pearson correlation (*r*) were performed.

#### Alteration in CBF in response to the HFM and WL.

To address the hypothesis of a significant underlying baseline CBF change in reward, appetite, and taste brain areas after HFM consumption, ASL CBF maps were formed at baseline and at 40 and 65 min postmeal (Figure 1) for the HFM and WL. Label and control ASL data sets were transformed to MNI space and surround-subtracted to yield a mean CBF image, which was spatially smoothed (8-mm full width at half maximum). CBF images were normalized by the baseline equilibrium magnetization and quantified in units of mL ⋅ 100 g^−1^ ⋅ min^−1^ with the use of the general kinetic model (21). To test for brain areas that showed a significant difference in gray matter CBF after the HFM or WL, CBF difference maps were formed by subtracting the 40- and 65-min postmeal maps from the before-meal consumption (baseline) maps, and areas significantly different from zero were displayed at a significance of *P* < 0.05 familywise error (22). In addition, at each time point, mean gray matter CBF was assessed in a priori areas of interest anatomically defined from the PickAtlas. This included the hypothalamus, thalamus, striatum, and insula cortex, with the visual cortex (middle occipital lobe) used as a control ROI (22, 23). The percentage change in gray-matter CBF in these ROIs at 40 and 65 min postmeal was plotted for the HFM and WL. In addition, to determine whether CBF changes underlie changes in BOLD activation, BOLD β-values to the CS and FS after the HFM and WL were plotted against the CBF change postmeal (mean: 40 and 65 min postmeal).

#### Effect of increasing subjective plasma CCK concentration after the HFM on the BOLD response.

To assess the relation between individual plasma CCK concentration and the combined BOLD response to the CS and FS, first-level CS and FS brain activation maps after the HFM were entered into an RFX 1-sample *t* test group analysis with the use of an individual subject’s plasma CCK concentration at 30 min postmeal as a covariate of interest. *P* ≤ 0.001 (uncorrected and corrected with the use of small-volume correction) was considered the threshold of significance for maps of brain areas that showed a positive and negative correlation with CCK concentration. An ROI analysis was also performed in a priori areas to illustrate the correlation of BOLD β-values to the CS and FS after the HFM with subject-specific CCK concentrations. A Spearman correlation (ρ) test was also performed.

For all analyses, PROP taster status was not included as a covariate in the model because a paired randomized design was used and because no significant difference was found in BOLD β-values between medium tasters and super tasters.

## Results

### 

Data from 1 woman were discarded because her head motion was >1 voxel during the fMRI scan. The remaining 16 subjects were 25 ± 2 y with a BMI of 22 ± 0.7.

#### Effect of HFM on subjective ratings and plasma CCK concentration.

**FIGURE 2:**
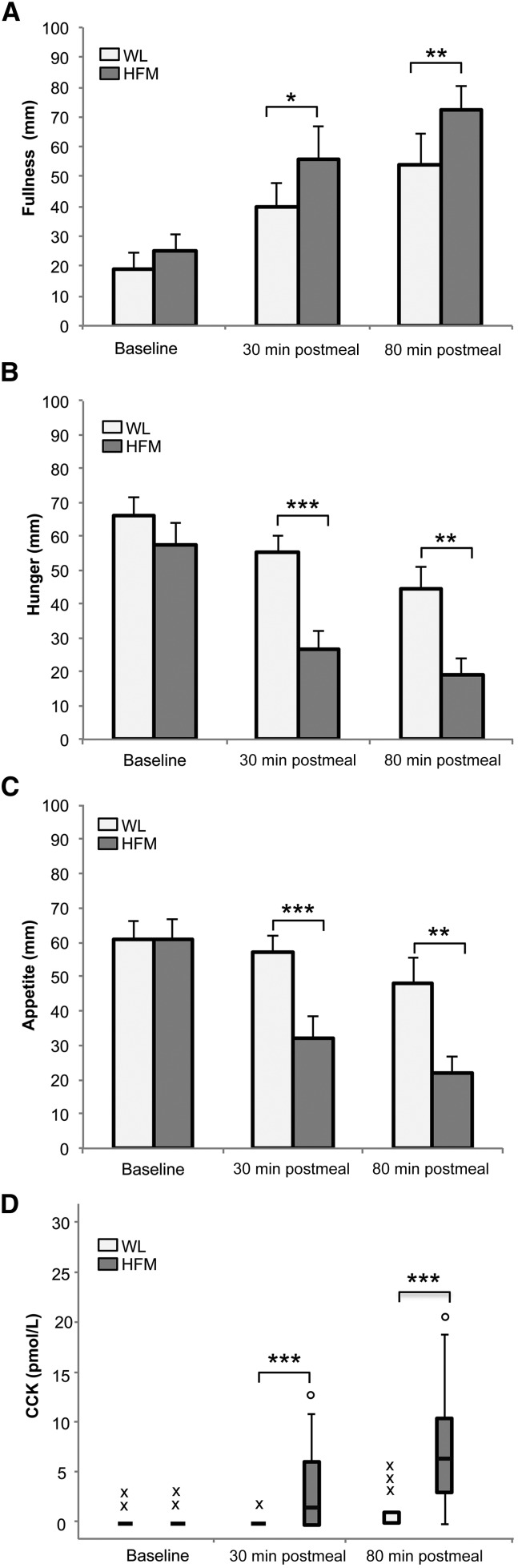
Subjective ratings of fullness (A), hunger (B), and appetite (C) and plasma CCK concentration (D) at baseline and at 30 and 80 min postmeal for HFM and WL in healthy adults. Data are means ± SEMs (A, B, and C), *n* = 16. Median, first, and third quartiles and minor and major outliers are shown in (D). Differences between WL and HFM: *P < 0.05, **P < 0.01, and ***P < 0.001. CCK, cholecystokinin; HFM, high-fat meal; WL, water load.

**Figure 2** shows subjective ratings of fullness, hunger, and appetite at baseline and at 30 and 80 min postmeal for the WL and HFM. At baseline, no significant difference in fullness, hunger, or appetite was seen between the WL and HFM. As expected, a significant increase in fullness (*P <* 0.05; *P <* 0.01) and a reduction in hunger (*P <* 0.001; *P <* 0.01) and appetite (*P <* 0.001; *P <* 0.01) were seen for the HFM at 30 and 80 min postmeal, respectively, compared to the WL. For the HFM, significant differences were found over the 3 time points for fullness (*P <* 0.001), hunger (*P <* 0.001), and appetite (*P <* 0.01). For the WL, only fullness (*P <* 0.001) and hunger (*P <* 0.05) showed significant differences across the 3 time points, with no significant differences in appetite.

As shown in Figure 2D, CCK concentration was significantly elevated after the HFM compared to the WL at 30 and 80 min postmeal (*P <* 0.001 and *P <* 0.001, respectively; no significant difference at baseline). After the HFM, CCK concentration increased significantly over the duration of the fMRI scan (*P <* 0.001). For the WL, no significant difference in CCK concentration was found at either 30 or 80 min postmeal compared to baseline, although CCK concentration did increase at 80 min postmeal as a result of the FS consumed during the fMRI scan. After the HFM, individual subjective ratings of fullness, hunger, and appetite correlated with individual subjects’ CCK concentrations across the 3 time points. A positive correlation of CCK concentration with fullness (ρ = 0.53; *P <* 0.001) and negative correlation of CCK concentration with hunger (ρ = −0.51; *P <* 0.001) and appetite (ρ = −0.40; *P <* 0.01) were found.

#### fMRI neural activation.

**FIGURE 3:**
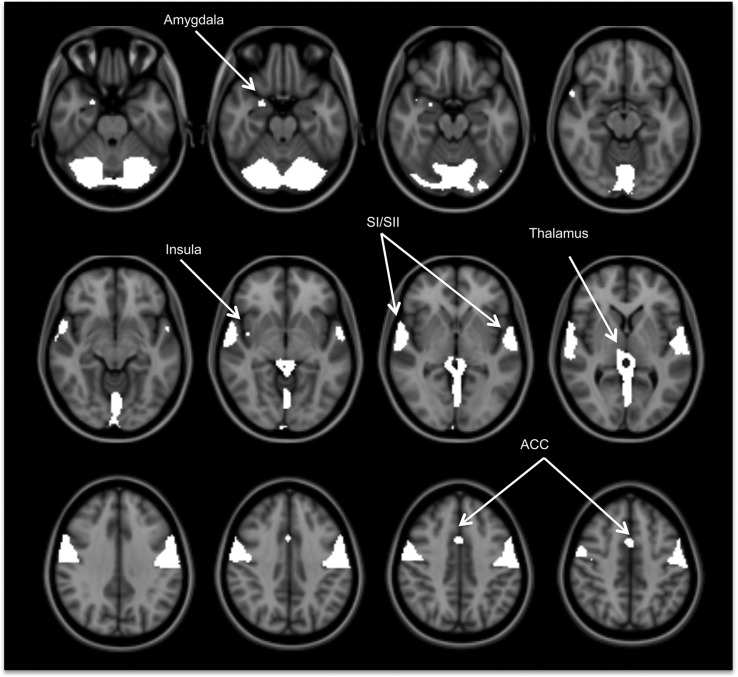
Activated brain regions in response to the fat stimulus after the high-fat meal in healthy adults, *n* = 16. The activation map had a false-discovery rate–corrected threshold of* P* < 0.05. ACC, anterior cingulate cortex; SI, primary somatosensory cortex; SII, secondary somatosensory cortex.

The CS and FS elicited robust BOLD activity in a large network of brain areas, including primary taste areas (anterior insula and frontal operculum), oral somatosensory areas (middle and posterior insula, SI, SII, and rolandic operculum), reward areas (ACC and amygdala), and the thalamus (**Figure 3**).

#### Effect of prior consumption of an HFM and WL on BOLD activation.

**FIGURE 4:**
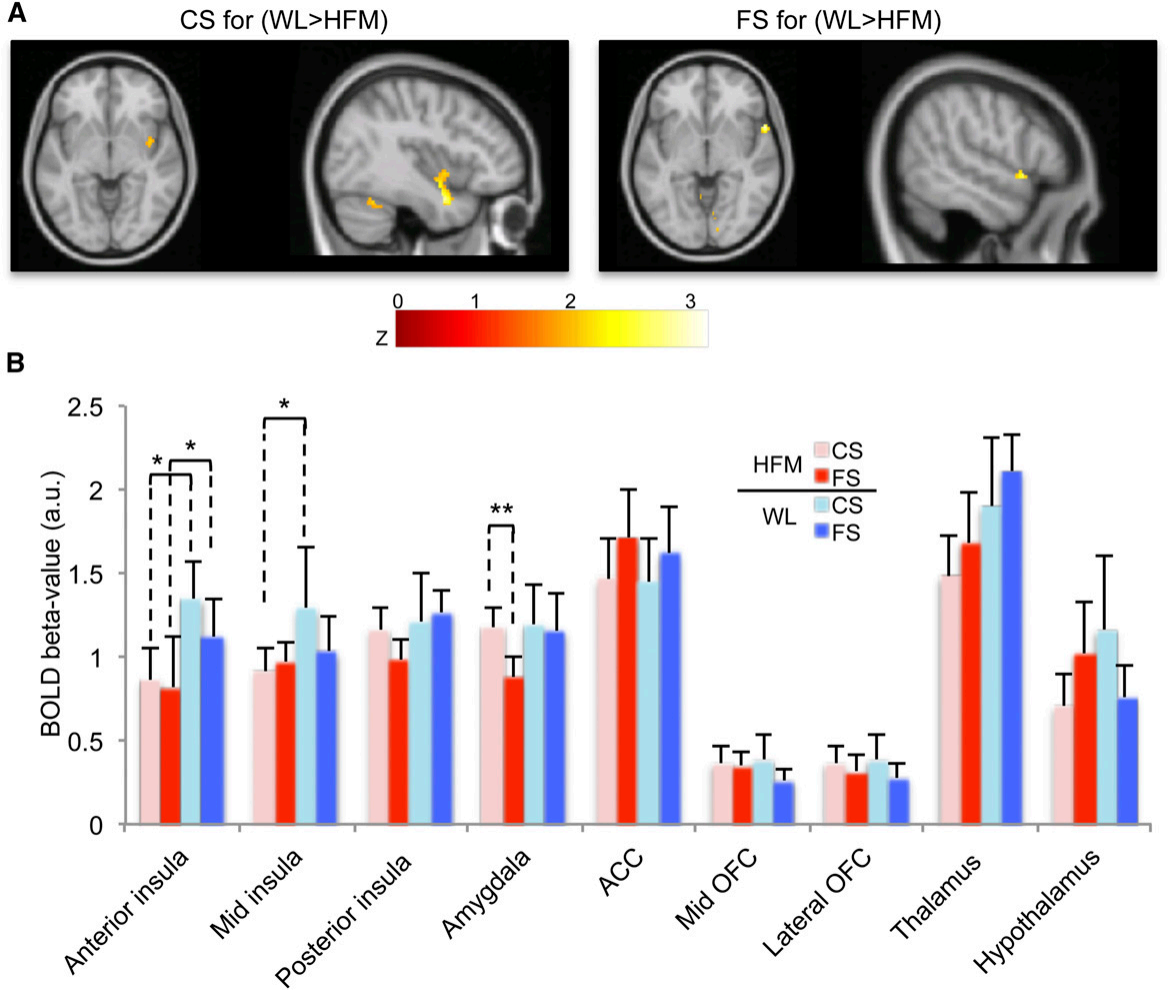
Decrease in BOLD response after the HFM in healthy adult subjects, *n* = 16. (A) Activation maps of the WL > HFM contrast for the CS and the FS displayed at* P* < 0.05 (uncorrected). (B) Mean ± SEM BOLD β-values (combined across hemispheres) in a priori cortical areas. *Difference between the HFM and WL for the CS and FS, *P* < 0.05. **Difference between the FS compared to the CS (fat-related satiety) for the HFM, *P* < 0.01. ACC, anterior cingulate cortex; a.u., arbitrary unit; BOLD, blood oxygenation level–dependent; CS, control stimulus; FS, fat stimulus; HFM, high-fat meal; OFC, orbitofrontal cortex; WL, water load.

When we compared the BOLD response to the CS and the FS after the consumption of an HFM and a WL, we observed that the HFM resulted in a decrease in the BOLD response to both the CS and FS in the bilateral anterior insula and frontal operculum compared to the WL (**Table 1**, **Figure 4A**). BOLD β-values in a priori ROIs (Figure 4B) confirmed this significant reduction in anterior (36.2%; *P <* 0.05) and midinsula (29.2%; *P <* 0.05) BOLD activation for the CS, and anterior insula (26.5%; *P <* 0.05) BOLD activation for the FS after the HFM. To assess fat-related satiety, we compared the BOLD response to the FS with the CS after both the HFM and WL. After the HFM, a significant reduction in amygdala activation (26.6%; *P <* 0.01) was found to the FS compared to that of the CS. Although not significant, a reduction in the posterior insula BOLD activation (15.7%; *P* = 0.07) and an increase in ACC activation (17.1%; *P* = 0.08) was also seen after the HFM when comparing the FS to the CS. After WL consumption, no significant difference in BOLD activation was found between the FS and CS.

**TABLE 1:** Prior HFM consumption compared to WL (WL > HFM) on the BOLD activation to the CS and FS in healthy adults^1^

	MNI^2^					
Area	*x*	*y*	*z*	*z* Score	*P* value	Cluster size^3^
CS						
Anterior insula RH	42	−2	−12	2.21	0.014	158
Anterior insula LH	−36	2	−22	2.04	0.002	14
FS						
Operculum/insula RH	56	12	−4	2.63	0.004	48
Operculum/insula LH	−60	6	10	2.68	0.015	18

1 Values are clusters of mean brain activation, *n* = 16. Clusters are brain areas that show a significantly greater decrease in BOLD activation after the HFM compared to the WL. BOLD, blood oxygenation level–dependent; CS, control stimulus; FS, fat stimulus; HFM, high-fat meal; LH, left hemisphere; MNI, Montreal Neurological Institute; RH, right hemisphere; WL, water load.

2 Peak voxel coordinates given in MNI space (*x*, *y*, *z*).

3 Reported clusters are at uncorrected-for-multiple-comparisons thresholds of *P* < 0.05, with a cluster-extent threshold >10 voxels.

#### Effect of prior consumption of an HFM and WL on the trial-by-trial BOLD response.

**FIGURE 5:**
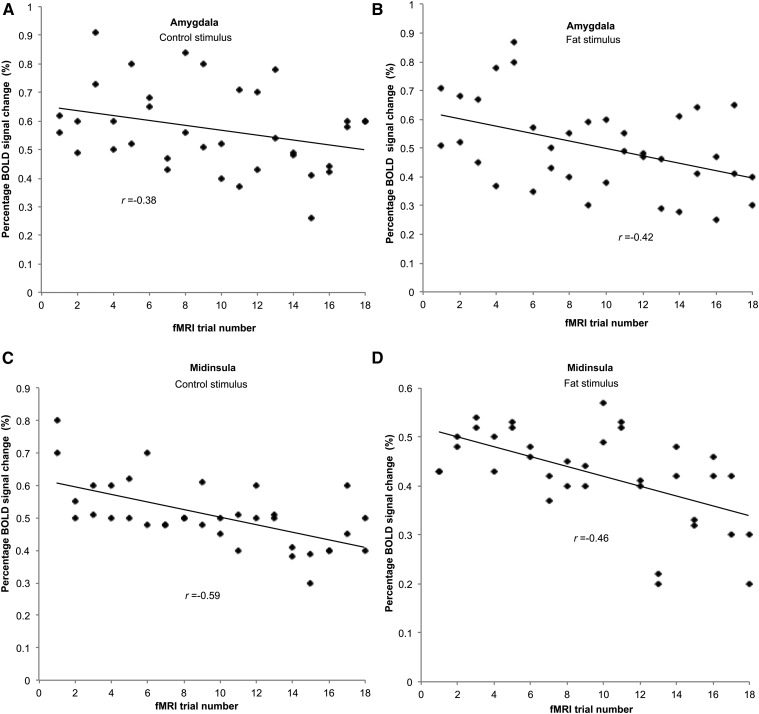
Habituation in BOLD percentage signal change after HFM consumption across the 18 CS and FS trials. Data are shown for the amygdala response to CS (A) and FS (B) and midinsula response to CS (C) and FS (D). Data are pooled from both hemispheres for each subject, *n* = 36. BOLD, blood oxygenation level–dependent; CS, control stimulus; FS, fat stimulus; HFM, high-fat meal.

In several areas, a significant reduction in the BOLD response to the CS and FS was found across trial number after the HFM (**Table 2**) and WL (**Table 3**). BOLD activation to the CS and FS across trial number was enhanced only in the bilateral precentral gyrus after the HFM [(42, −10, 36), *z* = 3.25, *P* < 0.001; (−40, −10, 36), *z* = 3.0, *P* < 0.001], and in the left prefrontal gyrus [(−32, 54, 24), *z* = 3.18, *P* < 0.001)] after the WL. **Table 4** summarizes the Pearson correlation coefficient (*r*) associated with the trial-by-trial reduction in the BOLD activation in a priori ROIs and shows the greater degree of habituation of BOLD activation after the HFM compared to the WL. **Figure 5** illustrates this trial-by-trial reduction in the BOLD percentage signal change in the amygdala and midinsula after the HFM.

**TABLE 2:** Habituation of BOLD activation to the CS and FS after the HFM in healthy adults^1^

	MNI^2^						
Area	*x*	*y*	*z*	*z* Score	*P* value	Cluster size^3^	SVC^4^
Operculum/insula RH	42	−8	−2	3.23	0.001	434	0.020
Amygdala RH	22	4	−22	3.43	<0.001	18	0.010
Temporal gyrus LH	−62	−30	16	3.98	<0.001	33	0.002
Operculum LH	−52	−18	14	3.93	<0.001	67	0.002
Precentral gyrus LH	−54	4	36	3.47	<0.001	24	0.010
Inferior parietal LH	−52	−32	52	4.27	<0.001	56	<0.000
Inferior frontal RH	60	8	4	3.36	<0.001	31	0.019

1 Values are clusters of mean brain activation, *n* = 16. Clusters are brain areas that show a habituation in BOLD activity across fMRI trials after the HFM for the CS and FS. BOLD, blood oxygenation level–dependent; CS, control stimulus; FS, fat stimulus; HFM, high-fat meal; LH, left hemisphere; MNI, Montreal Neurological Institute; RH, right hemisphere; SVC, small-volume correction.

2 Peak voxel coordinates given in MNI space (*x*, *y*, *z*).

3 Reported clusters are at an uncorrected-for-multiple-comparisons threshold of *P* < 0.001 (which corresponds to a *z* score >3), with a cluster-extent threshold >10 voxels.

4 Activations corrected by defining an 8-mm radius sphere around a peak voxel in a priori areas at *P* < 0.05.

**TABLE 3:** Habituation of BOLD activation to the CS and FS after the WL in healthy adults^1^

	MNI^2^						
Area	*x*	*y*	*z*	*z* Score	*P* value	Cluster size^3^	SVC^4^
Anterior insula RH	56	8	4	4.23	<0.001	181	0.001
ACC RH	6	4	52	3.72	<0.001	23	0.003
Supramarginal gyrus LH	−58	−30	52	3.65	<0.001	24	0.014
Temporal gyrus RH	60	−30	8	3.53	<0.001	89	0.002
Temporal gyrus LH	−60	2	−2	3.12	0001	14	0.027
Thalamus LH	−4	−8	6	3.14	0.001	20	0.005

1 Values are clusters of mean brain activation, *n* = 16. Clusters are brain areas that show a habituation in BOLD activity across fMRI trials after the WL for the CS and FS. ACC, anterior cingulate cortex; BOLD, blood oxygenation level–dependent; CS, control stimulus; FS, fat stimulus; LH, left hemisphere; MNI, Montreal Neurological Institute; RH, right hemisphere; SVC, small-volume correction; WL, water load.

2 Peak voxel coordinates given in MNI space (*x*, *y*, *z*).

3 Reported clusters are at an uncorrected-for-multiple-comparisons threshold of *P* < 0.001 (which corresponds to a *z* score >3), with a cluster-extent threshold >10 voxels.

4 Activations corrected by defining an 8-mm radius sphere around a peak voxel in a priori areas at *P* < 0.05.

**TABLE 4:** Correlation coefficients showing the decrease in BOLD activation (habituation) across fMRI trials in a priori brain ROIs in healthy adults^1^

	HFM		WL	
ROI	CS	FS	CS	FS
Amygdala	*−*0.38*	*−*0.42**	—	—
Anterior insula	—	—	—	*−*0.40*
Midinsula	*−*0.59**	*−*0.46**	—	—
Posterior insula	*−*0.44**	*−*0.41*	—	—
Thalamus	—	—	*−*0.38*	—

1 Values are Pearson correlations (*r*) of the decrease in BOLD activation across the 18 fMRI trials of the CS and FS after the HFM and WL in a priori brain ROIs, *n* = 16. *P < 0.05 and **P < 0.01. BOLD, blood oxygenation level–dependent; CS, control stimulus; FS, fat stimulus; HFM, high-fat meal; ROI, region of interest; WL, water load.

#### Alteration in CBF in response to the HFM and WL.

**FIGURE 6:**
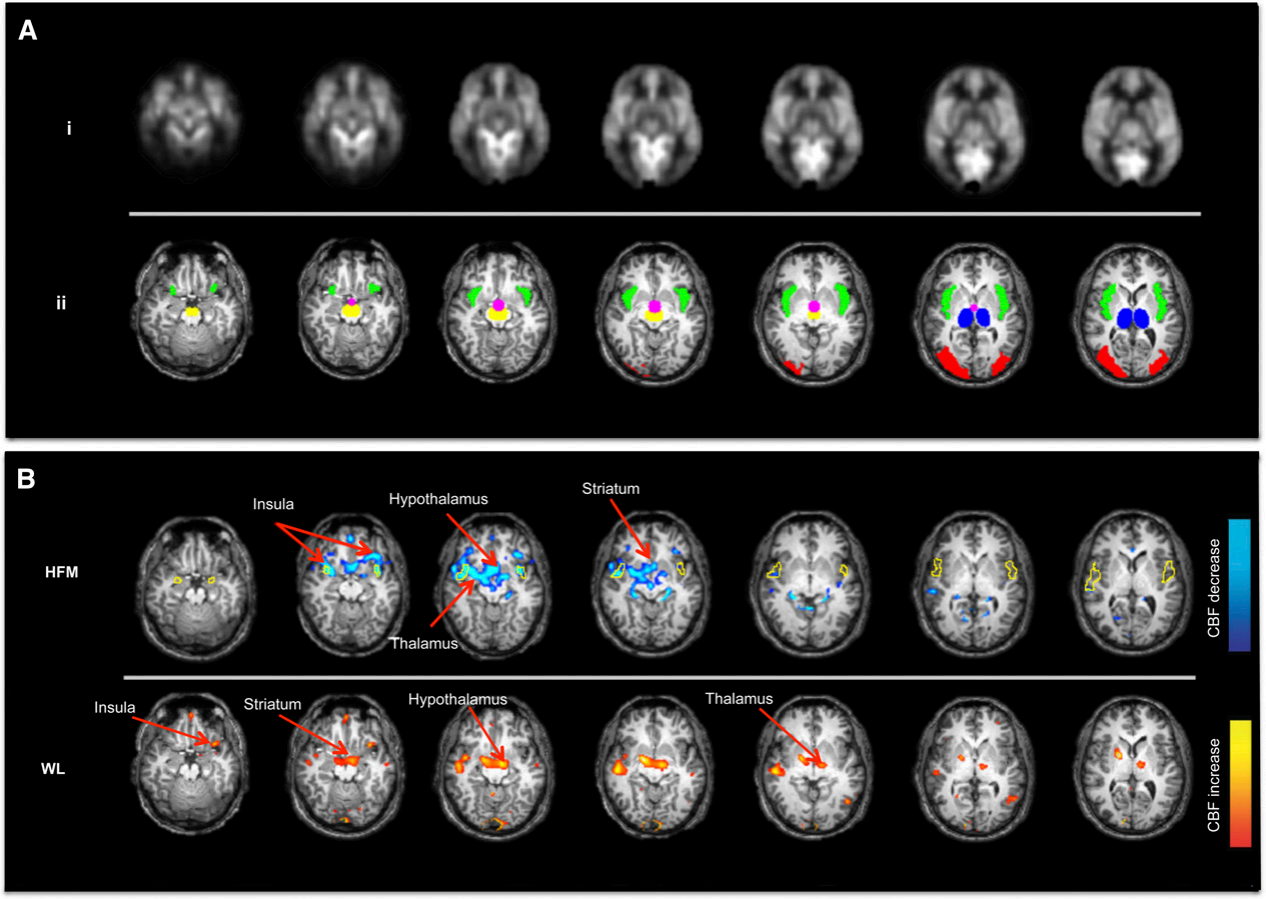
Change in CBF after the HFM and WL. (A) Baseline CBF image, *n* = 16 (i); ROIs interrogated in the CBF analysis: insula (green), hypothalamus (pink), thalamus (blue), striatum (yellow), and visual cortex (red) (ii). (B) Maps of the CBF change after the HFM (baseline to 65 min postmeal) and WL (baseline to 40 min postmeal). CBF was reduced (blue) in the hypothalamus, thalamus, striatum, and insula after the HFM. CBF was increased (orange) in the thalamus and insula after the WL. The yellow outline shows the insula BOLD response to the CS and FS as seen in Figure 3. Maps are displayed at *P* < 0.001 (uncorrected). BOLD, blood oxygenation level–dependent; CBF, cerebral blood flow; CS, control stimulus; FS, fat stimulus; HFM, high-fat meal; WL, water load.

The mean CBF image and brain ROIs interrogated (overlaid on a structural image) are shown in **Figure 6A**. Figure 6B shows brain areas with a significant difference in CBF postmeal compared to baseline, with data showing a significant CBF decrease for the HFM (baseline to 65 min postmeal) and increased CBF for the WL (baseline to 40 min postmeal). The HFM resulted in a significant CBF decrease in the hypothalamus, thalamus, insula, and striatum at 65 min postmeal; no such reduction in CBF was seen after the WL. In contrast, CBF was found to increase at 40 min postmeal after the WL in both the thalamus and insula (Figure 6B). No brain region showed a significant increase in CBF at 65 min postmeal after either the HFM or WL.

**FIGURE 7:**
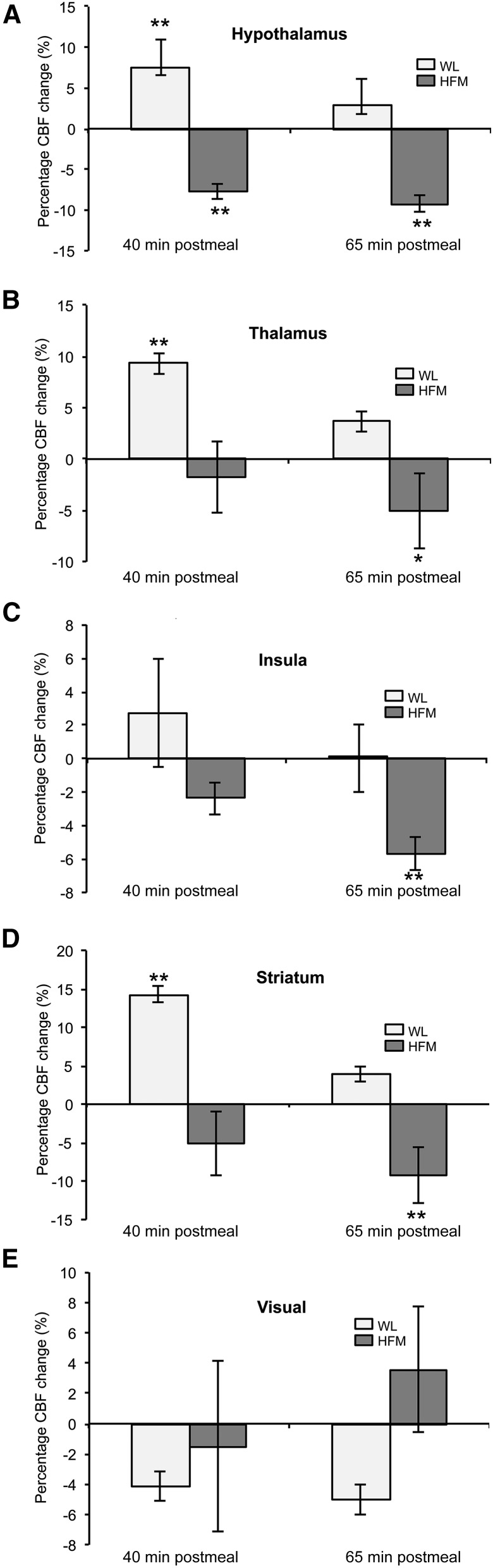
Mean ± SEM percentage change in CBF after the WL and HFM for hypothalamus (A), thalamus (B), striatum (C), insula (D), and visual cortex (E), *n* = 16. Different from baseline: *P < 0.05 and **P < 0.01. CBF, cerebral blood flow; HFM, high-fat meal; WL, water load.

**FIGURE 8:**
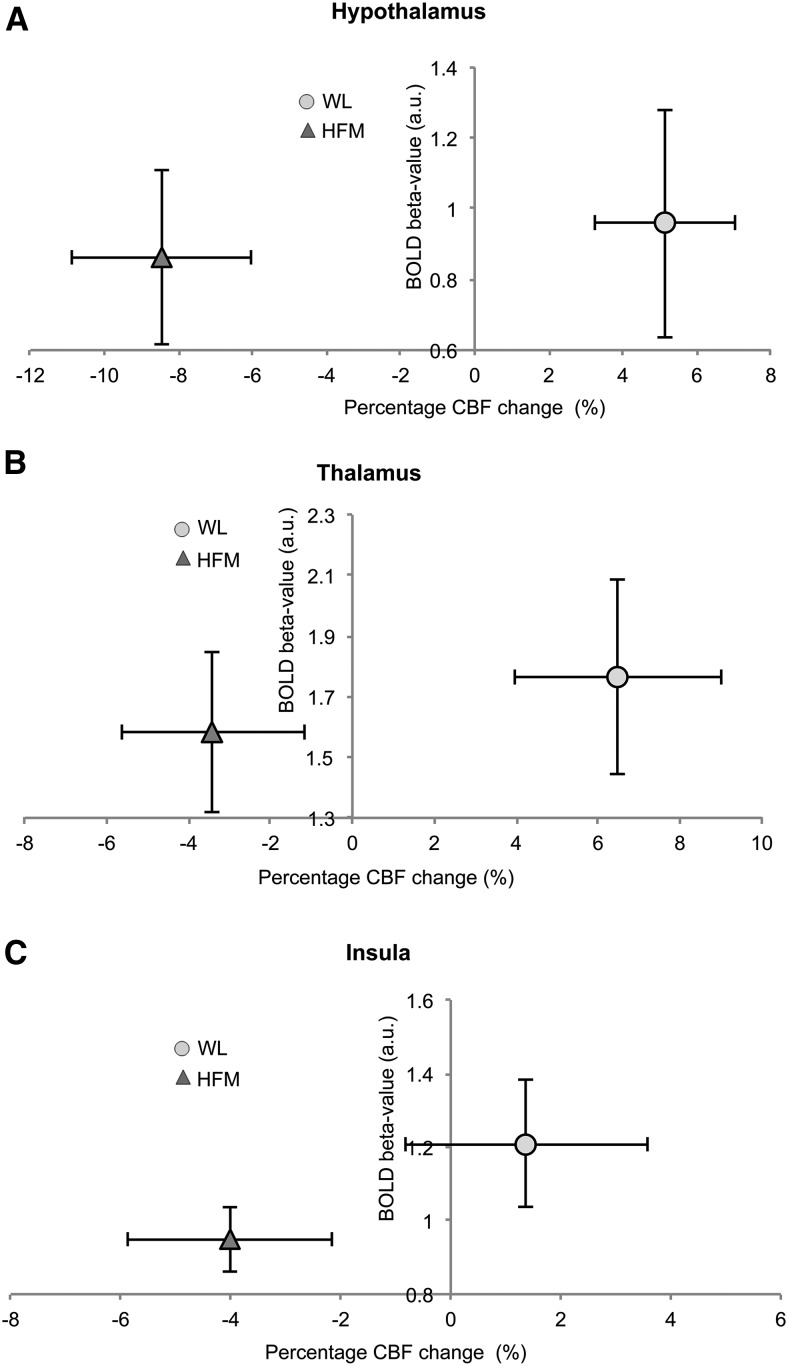
BOLD signal change to CS and FS after the WL and HFM plot against associated CBF percentage change (mean CBF at 40 and 65 min postmeal) for hypothalamus (A), thalamus (B), and insula (C). BOLD, blood oxygenation level–dependent; CBF, cerebral blood flow; CS, control stimulus; FS, fat stimulus; HFM, high-fat meal; WL, water load.

**Figure 7** provides the percentage change in CBF at 40 and 65 min postmeal compared to baseline in a priori ROIs. After the HFM, a significant reduction in CBF was found in the hypothalamus at both 40 and 60 min (7.7% and 9.2%, respectively; *P <* 0.01) postmeal, whereas CBF in the striatum (9.2%; *P <* 0.01), insula (5.7%; *P <* 0.01), and thalamus (5.1%; *P <* 0.05) reduced significantly at 65 min postmeal. In contrast, an increase in CBF was found after the WL at 40 min postmeal in the hypothalamus (7.5%; *P <* 0.01), striatum (14.3%; *P <* 0.01), and thalamus (9.3%; *P* < 0.01). In the visual cortex, no significant changes in CBF were found postmeal for either the HFM or WL. **Figure 8** shows for a priori ROIs the BOLD β-values to the CS and FS after the WL and HFM plot against the percentage CBF change after the WL and HFM (mean: 40 and 65 min postmeal). The plot shows that the reduction in insula BOLD response to the CS and FS after the HFM is driven by a reduction in baseline CBF.

#### Effect of increasing subjective plasma CCK concentration after the HFM on the BOLD response.

**FIGURE 9:**
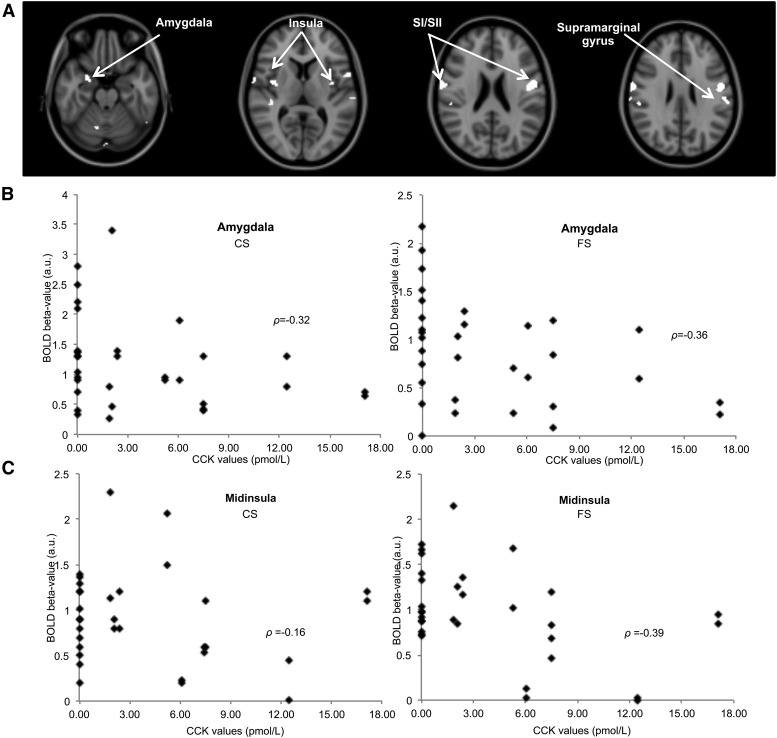
Negative correlation of the BOLD activation to the CS and FS with plasma CCK concentration after the HFM in individual healthy adult subjects; *n* = 16. (A) Brain regions in which individual’s BOLD response to the CS and FS after the HFM negatively correlated with plasma CCK concentration; maps are displayed at *P* < 0.005 (uncorrected). Scatter plots show increased plasma CCK concentration results in decreased BOLD β-values to the CS and FS after the HFM in the amygdala (B) and midinsula (C) (data points shown for each hemisphere and subject, *n* = 32). BOLD, blood oxygenation level–dependent; CCK, cholecystokinin; CS, control stimulus; FS, fat stimulus; HFM, high-fat meal; SI, primary somatosensory cortex; SII, secondary somatosensory cortex.

To evaluate associations between plasma CCK concentration and BOLD activity, an individual subject’s plasma CCK concentration was used as a covariate in the RFX analysis of the BOLD response to the CS and FS. We observed a significant decrease in this BOLD activation with increasing CCK concentration in the SI, anterior, middle, and posterior insula; amygdala; superior temporal gyrus; supramarginal gyrus; and cerebellum) (**Table 5**, **Figure 9A**). This significant negative correlation of BOLD activation to the CS and FS with CCK concentration is illustrated in a priori ROIs of the amygdala (*P <* 0.05; ρ* =* −0.36) and midinsula (*P <* 0.05; ρ* =* −0.39) in Figure 9B, C .

**TABLE 5:** Brain areas in which BOLD activation to the CS and FS after the HFM negatively correlates with CCK plasma concentration in healthy adults^1^

	MNI^2^						
Area	*x*	*y*	*z*	*z* Score	*P* value	Cluster size^3^	SVC^4^
SI RH	66	−4	24	3.59	<0.001	17	0.007
Supramarginal gyrus RH	66	−20	36	4.18	<0.001	36	0.003
Supramarginal gyrus LH	−58	−24	30	3.53	<0.001	21	0.011
Operculum/insula LH	−54	4	4	3.65	<0.001	20	0.006
Midinsula LH	−40	−2	−4	3.80	<0.001	36	0.004
Posterior insula RH	50	−30	18	4.03	<0.001	22	0.002
Amygdala RH	24	−2	−28	3.49	<0.001	32	0.018
Amygdala LH	−20	0	−32	3.51	<0.001	11	0.009
Operculum LH	−50	−4	22	3.50	<0.001	29	0.012
Temporal gyrus RH	44	−54	−36	3.42	<0.001	20	0.012
Cerebellum LH	−18	−68	−58	3.95	<0.001	12	0.025
Thalamus LH	−10	−16	6	4.64	<0.001	11	<0.000

1 Values are clusters of mean brain activation, *n* = 16. Clusters are those brain areas for which individual BOLD activation to the CS and FS after the HFM correlates negatively with the plasma CCK concentration. BOLD, blood oxygenation level–dependent; CCK, cholecystokinin; CS, control stimulus; FS, fat stimulus; HFM, high-fat meal; LH, left hemisphere; MNI, Montreal Neurological Institute; RH, right hemisphere; SI, primary somatosensory cortex; SVC, small-volume correction.

2 Peak voxel coordinates given in MNI space (*x*, *y*, *z*).

3 Reported clusters are at uncorrected-for-multiple-comparisons thresholds of *P* < 0.001 (which corresponds to a *z* score >3), with a cluster-extent threshold >10 voxels.

4 Activations corrected by defining an 8-mm radius sphere around a peak voxel in a priori areas at *P* < 0.05.

## Discussion

### 

We have shown that the prior consumption of an HFM leads to an increased CCK concentration and subjective ratings of fullness. BOLD anterior insula taste activation to both the CS and FS decreased after HFM consumption compared to the WL. The HFM caused reduced amygdala activation in response to the FS compared to the CS (fat-related satiety). Individual subjects’ plasma CCK concentrations negatively correlated with BOLD activation in taste and reward areas. This suggests that taste-, appetite-, and reward-related areas are responsive to nutritional status and, as such, receive not only sensory signals but also introspective signals of motivation and/or hedonic value.

#### Effect of prior consumption of an HFM and WL on BOLD activation.

We observed BOLD responses to the CS and FS in primary taste areas, oral somatosensory areas, reward areas, and the thalamus to be consistent with previous studies of fat emulsions and flavor studies (7, 19).

Prior consumption of the HFM attenuated the BOLD response to the CS and FS in the anterior insula primary taste area and midinsula oral somatosensory area compared to the WL. This suppression of anterior insula activity suggests that this region plays a role in feeding behavior in addition to its role in taste-sensory processing. The insula is an important relay that connects the hypothalamus, OFC, and limbic system. We postulate that the insula is selectively affected by the metabolic, hormonal, or neuronal events that signal the state of hunger or fullness, which has been shown to have a modulatory effect on the cortical response. This is consistent with previous studies of satiation to water (24), chocolate (25), tastants (16), and odor stimuli (13). The midinsula is an oral somatosensory area that responds to textural attributes of stimuli (6, 7). There was also a trend for an attenuation of the BOLD response to the FS in the posterior insula after the HFM compared to the WL; this trend is consistent with the postulation that it is related to autonomic function and gastric distention (26). The amygdala showed a significantly reduced BOLD response to the FS compared to the CS after the HFM, reflecting a fat-related satiety response, and a trend for a suppression of the BOLD response to the FS after the HFM compared to the WL. The amygdala has been implicated in the emotional processing of stimuli (27) and is thought to play an important role in feeding behavior. Prior neuroimaging studies have reported amygdala activation in response to taste and odor stimuli (7, 27) and suppression of satiation as a result of decreased reward (13, 15, 28). After the HFM compared to WL, there was also a nonsignificant decrease in the hypothalamus and thalamus BOLD response for the CS and conversely an increase in the hypothalamus BOLD response to the FS. Prior studies have shown that the hypothalamus is modulated by satiety (15, 29). Smeets et al. (15) reported a decrease in hypothalamus BOLD activation in response to satiation in women but not in men. The inconsistency of hypothalamus group activation may result from its small structure, positional variability, and close proximity to a sinus cavity. The reduction in thalamus activity is consistent with previous studies that have suggested that the thalamus integrates and relays sensory information to the cortex, potentially to regulate food intake (25).

In this study, the HFM was consumed orally, so we could not deconstruct the oral and gastrointestinal influences of the HFM. A design with a third arm that included subject intubation to deliver the HFM directly to the stomach was considered, but it was deemed too onerous for volunteers and likely would have led to a high risk of dropout from the full study. However, such work has been demonstrated by Spetter et al. (12), who showed an increase in brain activation in the midbrain, amygdala, hypothalamus, and hippocampus during gastric infusion of both a high-caloric meal and WL. In addition, areas involved with taste and reward processing have shown greater activation during oral consumption of the same meal than infusion (12), highlighting the importance of sensory stimulation in the satiation process.

#### Effect of prior HFM and WL consumption on the trial-by-trial BOLD response.

After HFM consumption, a significant reduction in the BOLD response to the FS across fMRI trials was seen in the amygdala and middle and posterior insula. Such variation in amygdala responses has previously been reported for olfactory (30) and taste (31) stimuli, and BOLD insula activation in response to water and sucrose stimuli has been shown to be significantly different between the first and second half of an fMRI paradigm (31). In agreement with Smeets et al. (15), the gradual enhancement of the BOLD response in the precentral gyrus to both the CS and FS likely reflects enhancing satiety signals.

#### Alteration in CBF in response to the HFM and WL.

After the HFM, a reduction in CBF was found in the hypothalamus, thalamus, striatum, and insula compared to baseline. Reduced CBF to these areas likely underlies the reduction in the BOLD response to the CS and FS after the HFM. The reduction in CBF in these areas is in line with a previous positron emission tomography study (32) that reported a negative correlation between plasma insulin (satiety stimulator) concentration and CBF in the insula. In addition, with the use of fMRI, Frank et al. (23) showed a pronounced reduction in CBF in the hypothalamus and insula after the consumption of high-fat yogurt compared to baseline; furthermore, in a given individual, they showed a significant correlation between the percentage CBF reduction in the hypothalamus and insula, indicating the functional interaction of these brain areas.

In this study, we also showed an increase in CBF in the thalamus and insula 40 min postmeal after the WL compared to baseline, which subsequently returned to baseline at 65 min postmeal. This increase at 40 min postmeal may reflect the opposing mechanism of reward from the WL because subjects fasted before the fMRI study.

#### Effect of increasing subjective plasma CCK concentration after the HFM on the BOLD response.

Several brain areas showed a negative correlation of BOLD activation to the CS and FS with individual subjects’ CCK plasma concentrations, including the SI, SII, anterior, middle, and posterior insula; amygdala; superior temporal gyrus; supramarginal gyrus; precentral gyrus; and cerebellum. Previous work has shown that an infusion of CCK (33) or consumption of an HFM (34) produces an elevation of CCK concentrations and greater feelings of satiety. The decrease of BOLD response to the CS and FS in the amygdala with increasing CCK concentrations herein supports a decrease in reward response resulting from satiation. The amygdala represents both the pleasantness and aversiveness of taste and olfactory stimuli (35) and has been shown to be involved in the homeostatic regulation of energy intake. Anterior and middle insula activity has previously been reported to be modulated by satiation (13, 24, 25), supporting the idea that the anterior and middle insula play an important role in food intake, whereas the increase of thalamus activity with increasing CCK concentrations may reflect an anorectic response (10).

In conclusion, prior HFM consumption increases the gut peptide hormone CCK and modulates the BOLD response to a subsequent FS or CS, with a greater habituation to the FS in reward, taste, and oral somatosensory areas and enhancement of activation in the precentral gyrus, an area thought to be responsible for satiation. This habituation of the BOLD response is associated with an underlying reduction in CBF in the hypothalamus, thalamus, and insula, areas associated with reward and homeostatic function. Understanding the mechanism of oral fat satiation and perception will help inform the reformulation of reduced-fat foods that mimic the brain’s response to high-fat counterparts and guide future interventions to reduce obesity.

## Footnotes

13 Abbreviations used: ACC, anterior cingulate cortex; ASL, arterial spin labeling; BOLD, blood oxygenation level–dependent; CBF, cerebral blood flow; CCK, cholecystokinin; CS, control stimulus; EPI, echo-planar imaging; FS, fat stimulus; GE, gradient echo; HFM, high-fat meal; OFC, orbitofrontal cortex; MNI, Montreal Neurological Institute; PROP, 6-*n-*propylthiouracil; RFX, random effects; ROI, region of interest; SI, primary somatosensory cortex; SII, secondary somatosensory cortex; TE, echo time; TR, repetition time; WL, water load.
